# The Effects of Acupuncture at Real or Sham Acupoints on the Intrinsic Brain Activity in Mild Cognitive Impairment Patients

**DOI:** 10.1155/2015/529675

**Published:** 2015-05-03

**Authors:** Baohui Jia, Zhishun Liu, Baoquan Min, Zhenchang Wang, Aihong Zhou, Yong Li, Haifa Qiao, Jianping Jia

**Affiliations:** ^1^Department of Acupuncture, Guang'anmen Hospital, China Academy of Chinese Medical Sciences, Beijing 100053, China; ^2^Department of Neurology, Xuanwu Hospital of Capital Medical University, Beijing 100053, China; ^3^Department of Biomedical Sciences, Florida State University College of Medicine, Tallahassee, FL 32306, USA; ^4^Department of Radiology, Tongren Hospital of Capital Medical University, Beijing 100730, China; ^5^Institute of Acupuncture and Moxibustion, China Academy of Chinese Medical Sciences, Beijing 100700, China

## Abstract

Accumulating neuroimaging studies in humans have shown that acupuncture can modulate a widely distributed brain network in mild cognitive impairment (MCI) and Alzheimer's disease (AD) patients. Acupuncture at different acupoints could exert different modulatory effects on the brain network. However, whether acupuncture at real or sham acupoints can produce different effects on the brain network in MCI or AD patients remains unclear. Using resting-state fMRI, we reported that acupuncture at Taixi (KI3) induced amplitude of low-frequency fluctuation (ALFF) change of different brain regions in MCI patients from those shown in the healthy controls. In MCI patients, acupuncture at KI3 increased or decreased ALFF in the different regions from those activated by acupuncture in the healthy controls. Acupuncture at the sham acupoint in MCI patients activated the different brain regions from those in healthy controls. Therefore, we concluded that acupuncture displays more significant effect on neuronal activities of the above brain regions in MCI patients than that in healthy controls. Acupuncture at KI3 exhibits different effects on the neuronal activities of the brain regions from acupuncture at sham acupoint, although the difference is only shown at several regions due to the close distance between the above points.

## 1. Introduction

Mild cognitive impairment (MCI) is defined as a slight impairment in cognitive function (typically memory) with otherwise normal function in the performance of activities of daily living. It is now widely accepted that MCI is a transitional phase between normal function and Alzheimer's disease (AD), during which cognitive impairment is progressing [1, 2]. Most recent findings suggest that sensitive neuroimaging and network analysis may play a special role in understanding the pathophysiological mechanism of MCI and AD. Several resting-state fMRI studies have investigated the neuronal integrity in the brain of the AD or MCI patients by different methods. Using resting functional magnetic resonance imaging (fMRI), Biswal et al. [3] were the first to demonstrate that spontaneous low-frequency fluctuations (LFFs; <0.08 Hz) of the blood oxygen level dependent (BOLD) signal during rest were of physiological importance. It suggested that regional spontaneous BOLD fluctuations likely reflect spontaneous neuronal activity [3–5]. Recently, this technique has been used to investigate the intrinsic or spontaneous brain activity in subjects with AD or MCI [6–15].

Previous studies have identified that acupuncture, as a therapy of traditional Chinese medicine (TCM), remains promising to treat neurological diseases including chronic pain, drug addiction, stroke, and dementia [16–18]. Now neuroimaging, in particular fMRI, is a versatile tool which has been applied to investigate the mechanisms of acupuncture. Accumulating neuroimaging studies in humans have shown that acupuncture can modulate a widely distributed brain network [19–29]. Acupuncture could activate the temporal lobe (such as hippocampus, insula), some regions of the parietal lobe, and cerebellum in AD patients [30]. These regions are consistent with impaired brain areas in AD patients, which are closely correlated with the cognitive function (memory, reason, language, executive, etc.). This study also provides the preliminary neurophysiological evidence for the potential effect of acupuncture on AD [31]. On the other hand, acupuncture at different acupoints could exert different modulatory effects on the brain network [20]. However, whether acupuncture at real or sham acupoints can produce different effects on the brain network in MCI or AD patients still remains elusive.

Here we used resting-state fMRI to investigate the effects of acupuncture at KI3 or sham acupoint which is 25 mm above Taixi (KI3) on brain regions in MCI patients. Our analyses established that acupuncture at KI3 or sham acupoint could produce effects on some brain regions which are components of default cognitive network in MCI patients, and the statistical difference was only exhibited in several regions.

## 2. Materials and Methods

### 2.1. Participants

Twenty-three right-handed subjects participated in this study. 8 MCI patients (2 males and 6 females, average age: 74.1 ± 7.8 years) and 15 elder healthy controls (8 males and 7 females, mean age: 73.7 ± 7.3 years) were recruited from Xuanwu Hospital affiliated to Capital Medical University, Beijing. All the patients received medical interview, physical examination, blood tests, brain magnetic resonance imaging (MRI), and neuropsychological assessment, including Mini-Mental State Examination (MMSE) [32], Alzheimer's Disease Assessment Scale-cognitive subscale (ADAS-cog) and clinical dementia rating (CDR) [33], modified Hachinski ischemic scale (HIS) [34], activity of daily living scales (ADL), and Hamilton depression scale (HDS), and met the following criteria: (a) impaired memory performance on a normalized objective verbal memory delayed-recall test; (b) normal or near-normal performance on global cognitive tests; (c) MMSE score >24; (d) intact activities of daily living; (e) global rating of 0.5 on the CDR scale, with a score of at least 0.5 on the memory domain; and (f) absence of dementia. The controls were identified as healthy through a regular health examination, MMSE, a clinical memory scale (CMS), HDS, and MRI and met the following criteria: (a) no neurological or psychiatric disorders such as stroke, depression, and epilepsy; (b) no neurological deficiencies such as visual or hearing loss; (c) no abnormal findings such as infarction or focal lesion in conventional brain MR imaging; (d) no cognitive complaints; (e) MMSE score of 28 or higher; (f) CDR score of 0. Participants with contraindications for MRI such as pacemaker, cardiac defibrillator, implanted material with electric or magnetic system, vascular clips or mechanical heart valve, cochlear implant, or claustrophobia were excluded. In addition, patients with a history of stroke, trauma, psychiatric disorders, drug/alcohol abuse, severe hypertension, diabetes, anemia, systematic diseases, and intellectual disability were also not included. Informed consent was obtained from each subject or his (her) guardian. This study was approved by the Medical Research Ethics Committee of Xuan Wu Hospital.

### 2.2. Data Acquisition

Data acquisition was performed on a GE 1.5-T Magnetom Sonata system using a standard head coil. Foam padding and headphones were used to limit head motion and reduce scanner noise. Structural images were acquired using a sagittal magnetization-prepared rapid gradient echo (MPRAGE) three-dimensional T1-weighted sequence (repetition time (TR) = 1970 ms, echo time (TE) = 3.9 ms, inversion time (TI) = 1100 ms, flip angle (FA) = 20°, and field of view (FOV) = 220 × 220). Subjects were instructed to keep their body frozen and eyes closed but not think of anything and fall asleep in particular during rest fMRI scanning. Functional images were collected by using an echo-planar imaging (EPI) sequence sensitive to BOLD contrast (TR = 2000 ms, TE = 30 ms, FA = 90°, and FOV = 220 × 220). Whole-brain volumes were acquired with 24 contiguous 5-mm thick transverse slices, with a 1 mm gap and in-plane resolution = 64 × 64, 180 volumes. Through a simple questionnaire after the scan, we knew that all the subjects were awake with their eyes closed during the fMRI scanning session.

### 2.3. Data Preprocessing

Image preprocessing was carried out using the Statistical Parametric Mapping (SPM5) package (http://www.fil.ion.ucl.ac.uk/spm). The first five volumes were discarded to allow for T1 equilibration effects and the adaptation of the subjects to the circumstances, and then all functional images were corrected for different intravolume acquisition times between slices using the sinc interpolation and for the intervolume geometrical displacement due to head movement using a six-parameter (rigid body) spatial transformation [35]. After the corrections, the images were normalized into the stereotaxic space [36] using an optimum 12-parameter affine transformation and nonlinear deformations [37] and then resampled to 3 mm isotropic voxels. Finally, all normalized data were further temporally band-pass-filtered (0.01–0.08 Hz) to reduce the effects of low-frequency drift and high-frequency physiological noises. One male healthy control (male) was excluded because of excessive movement (>2 mm of translation or 2° of rotation in any direction).

The functional datasets of all patients and healthy controls were preprocessed using the following main steps. (a) Slice timing: the differences of slice acquisition times of each individual were corrected using slice timing. (b) Realigning: the temporal processed volumes of each subject were realigned to the first volume to remove the head motion. All participants had less than 3 mm of translation in *x*-, *y*-, or *z*-axis and 3° of rotation in each axis. (c) Spatial normalization: the realigned volumes were spatially standardized into the MNI (Montreal Neurological Institute) space by normalizing with the EPI template via their corresponding mean image. Then, all the normalized images were resliced by 3.0 ∗ 3.0 ∗ 3.0 mm^3^ voxels. (d) Smoothing: Analysis of Functional Neuroimages (AFNI) software (http://afni.nimh.nih.gov) was used to filter linear drift. The normalized functional series were smoothed with a Gaussian kernel of 6 mm full width at half-maximum (FWHM).

### 2.4. Acupuncture

In this study, we selected KI3 which is one of the most frequently used acupoints for the treatment of impaired memory and locates in the depression between the tip of the medial malleolus and Achilles' tendon [38], as a real acupoint, and a point which is 25 mm directly above KI3 as a sham control. The acupuncture needles (0.25 × 40 mm, Suzhou Hwato Medical Instruments, China) were inserted bilaterally, vertically to a depth of about 20 mm and rotated right then left, at a frequency of 2 Hz for 60 s. During fMRI scanning, the needles were kept in the points.

### 2.5. Statistical Analysis

Using global assessment of random effect, the data of each group was linked and then analyzed statistically. One-sample *t*-test was used between resting state (baseline) and resting state with acupuncture at true or sham acupoints in each group. Two-sample *t*-test was performed between different groups (*P* < 0.01 (multiple correction)). The connected voxels greater than 30 were considered to be activated brain regions. The mean activated image was transformed to Talairach axis and superimposed three-dimension Talairach template in AFNI software [36] for the final localization of the activated region.

## 3. Results

### 3.1. Demographic and Neuropsychological Characteristics

Demographic and neuropsychological characteristics were shown in Table 1. There were no significant differences between groups in gender, age, and years of education. ADS-cog score in MCI is significantly different compared with that in control, whereas no significant difference was found in MMSE score between groups.

### 3.2. Amplitude of Low-Frequency Fluctuation (ALFF) Analysis within Group

**Figure 1:**
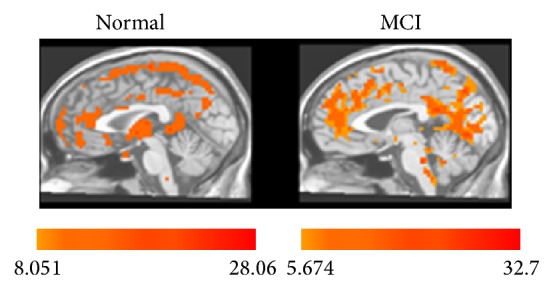
Mean ALFF maps in normal controls and MCI patients in the resting state. Color bar represents *t* value which increases with the darker color. The number under the color bar is the range of *t* value.

**Figure 2:**
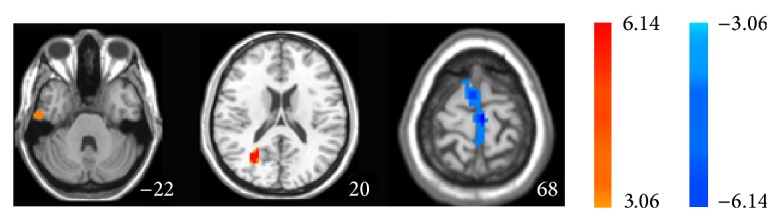
Unpaired *t*-test shows statistical difference map of ALFF between MCI patients and normal controls in the resting state.

Firstly we performed one-sample *t*-test to explore the ALFF patterns within group on the individual ALFF maps for each group. As shown in Figure 1, visual inspection indicated that frontal, parietal, and temporal lobes exhibited the increased ALFF values which are parallel with the distribution of default brain networks within each group. Narrowly, the increased regions include prefrontal lobe, posterior cingulate cortex, and precuneus.  Figure 2 and Table 2 showed the two-sample unpaired *t*-test of ALFF between the MCI patients and the healthy controls. In the resting state, ALFF value in MCI group was decreased significantly in the left medial frontal gyrus and was significantly increased in the right inferior temporal gyrus and posterior cingulate, comparing to the healthy group.

### 3.3. ALFF Analysis within Each Group in the Resting State Combined with Acupuncture at the Real Point

**Figure 3:**
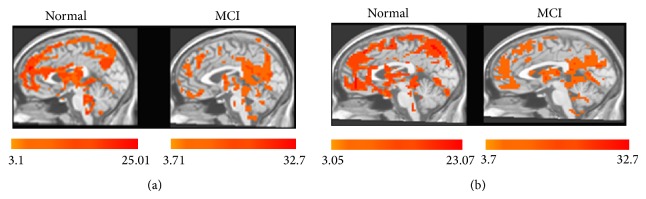
Mean ALFF maps in the resting state combined with acupuncture at real or sham acupoint in normal controls and MCI patients. (a) Mean ALFF maps within normal controls and MCI patients in the resting state combined with acupuncture at KI3. (b) Mean ALFF maps within normal controls and MCI patients in the resting state combined with acupuncture at sham acupoint.

Secondly, we explored the activated regions by acupuncture at real or sham acupoint in each group. As shown in Figures 3(a) and 3(b), regardless of acupuncture at real or sham acupoint, prefrontal, parietal, and temporal lobes displayed increased ALFF values within either MCI group or healthy controls in the resting state.

### 3.4. ALFF Analysis between Groups in the Resting State Combined with Acupuncture at the Real or Sham Acupoint

**Figure 4:**
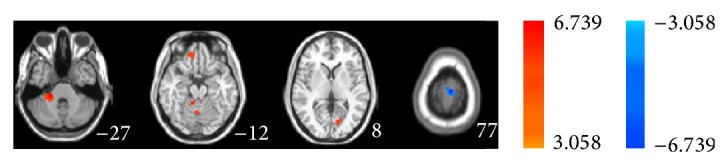
Unpaired *t*-test exhibits statistical difference map of ALFF between MCI patients and normal controls in resting state combined with acupuncture at KI3.

**Figure 5:**
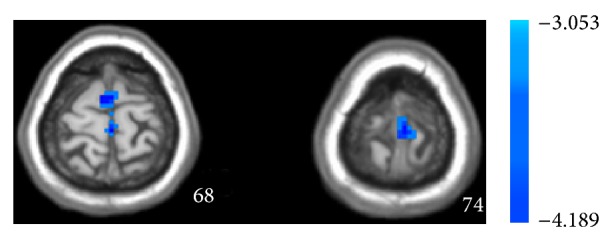
Unpaired *t*-test shows significant difference map of ALFF between MCI patients and normal controls in resting state combined with acupuncture at sham acupoint.

Subsequently we used two-sample unpaired *t*-test to analyze the difference between groups in the real or sham acupoint. As shown in Figure 4 and Table 3, in MCI patients, the regions showing decreased ALFF are postcentral gyrus, right medial frontal gyrus, cerebellar tonsil culmen, brainstem culmen, and left cuneus displaying increased ALFF in the resting state combined with acupuncture at the real point, comparing to the healthy controls. As shown in Figure 5 and Table 4, in MCI patients, acupuncture at sham acupoint caused tiny difference in the regions showing decreased ALFF between groups and only the left medial frontal gyrus and right superior frontal gyrus decreased ALFF value comparing to the healthy controls, suggesting that, in resting state, acupuncture at real and sham acupoints could activate different brain regions in both MCI and healthy groups.

### 3.5. ALFF Analysis between Resting State with and without Acupuncture at the Real Acupoint in MCI Patients

**Figure 6:**
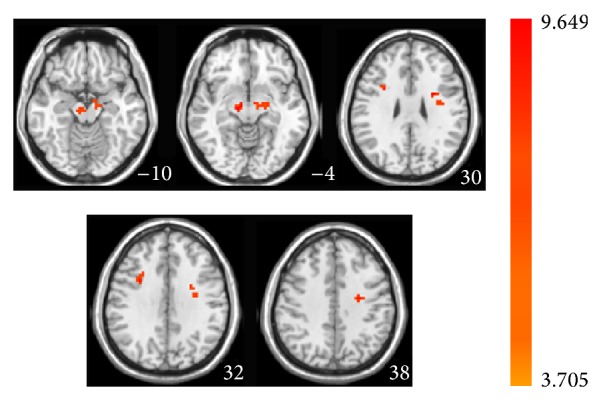
Two-sample paired *t*-test shows statistical difference map of ALFF in MCI patients between acupuncture at KI3 and no acupuncture in the resting state.

To explore the effect of acupuncture at the real acupoint on the ALFF in MCI patients, we performed the two-sample paired *t*-test to compare the ALFF between resting state without and with acupuncture. As shown in Figure 6 and Table 5, in MCI patients, acupuncture at KI3 in the resting state increased ALFF in the left parahippocampal gyrus, cingulate gyrus, middle frontal gyrus, right middle frontal gyrus, and subthalamic gyrus. In the healthy controls, acupuncture at the KI3 in the resting state increased ALFF in the right parahippocampal gyrus and reduced it in the right paracentral lobule and cuneus (see Figure  S1 and Table  S1 of the Supplementary Material available online at http://dx.doi.org/10.1155/2015/529675).

### 3.6. ALFF Analysis between Resting State with and without Acupuncture at the Sham Acupoint in MCI Patients

**Figure 7:**
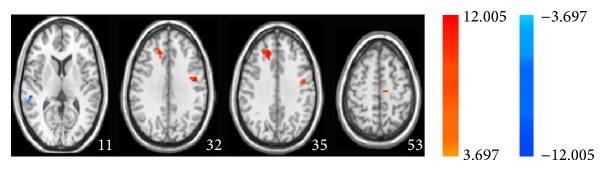
Two-sample paired *t*-test displays statistical difference map of ALFF in MCI patients between acupuncture at sham point and no acupuncture in the resting state.

Figure 7 and Table 6 showed the effect of acupuncture at the sham acupoint on the ALFF between resting states without and with acupuncture in MCI patients. Comparing to the resting state without acupuncture, acupuncture at the sham acupoint in the resting state enhanced ALFF in the left precentral gyrus, both right and left medial frontal gyrus, and decreased in the right superior temporal gyrus. In healthy controls, acupuncture at the sham acupoint in the resting state reduced the ALFF in the right cuneus, paracentral lobule but activated it in the right medial frontal gyrus, left anterior cingulate, and parahippocampal gyrus (Figure S2 and Table S2) comparing to the resting state without acupuncture. These data suggest that comparing to the resting state without acupuncture, acupuncture at the sham acupoint in MCI patients activated the different brain regions from those in healthy controls.

### 3.7. ALFF Analysis between Acupuncture at the Real and the Sham Acupoints in the Resting State

**Figure 8:**
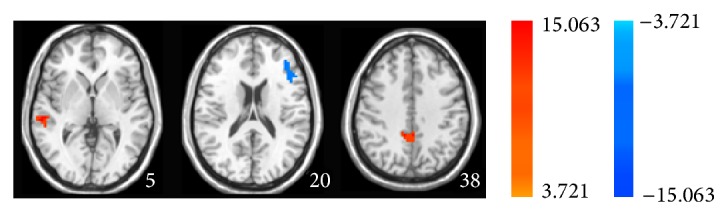
Two-sample paired *t*-test exhibits statistical difference map of ALFF in MCI patients between acupuncture at KI3 acupuncture and acupuncture at sham acupoint in the resting state.

Here a question was raised: what is the difference between acupuncture at the real and the sham acupoints? To answer this question, we conducted a two-sample paired *t*-test. As shown in Figure 8 and Table 7, in MCI patients, acupuncture at the KI3 increased ALFF in the right superior temporal gyrus, cingulate gyrus and decreased it in the middle front gyrus comparing to acupuncture at the sham acupoint in the resting state. In healthy controls, acupuncture at the real acupoint only decreased the ALFF in the left anterior cingulate comparing to acupuncture at the sham acupoint in the resting state (Figure S3 and Table S3). These data suggest that in the resting state acupuncture at KI3 can produce different effects on certain brain regions from acupuncture at the sham acupoint, especially in MCI patients.

## 4. Discussion

In the current study, we performed fMRI to detect the effects of acupuncture at KI3 and the sham acupoints on the resting-state brain activity in MCI patients and healthy controls and discovered that during the resting state brain activities in MCI patients were different from those of healthy subjects. In resting state combined with acupuncture, although acupuncture at KI3 or sham acupoint could not induce significant ALFF value change within either MCI group or healthy control, acupuncture at the real acupoint induced ALFF change of different brain regions in MCI patients from those shown in the healthy controls; acupuncture at sham acupoint also caused difference in MCI patients comparing to the healthy controls, suggesting that, in resting state, acupuncture at KI3 and sham acupoints could activate different brain regions in both MCI and healthy groups. We also found that, in MCI patients, acupuncture at KI3 in the resting state increased or decreased ALFF in the different regions from those activated by acupuncture in the healthy controls. In resting state, acupuncture at the sham acupoint in MCI patients activated the different brain regions from those in heath controls. Acupuncture at the KI3 can produce different effects on a few of brain regions in the resting state from acupuncture at the sham acupoint, especially in MCI patients.

The frontal and temporal regions were considered as important components of human default-mode networks [28–30] and have been shown to exhibit AD- and MCI-related structural and functional abnormalities [29]. By measuring the amplitude of the spontaneous activities (ALFF), several researchers have shown altered baseline brain activity in children with attention deficit hyperactivity disorder [39] and trauma survivors shortly after traumatic events [40]. Two recent resting fMRI studies also utilized ALFF to investigate the brain's spontaneous activity under eyes-open and eyes-closed conditions and found that the activities within the visual cortex and default-mode regions were significantly different between the two conditions [41, 42]. These recent studies indicate that the ALFF is physiologically meaningful for measuring intrinsic or spontaneous neuronal activity of the brain. In the present study, we found that in the resting state the ALFF of medial frontal gyrus decreased, but inferior temporal gyrus and posterior cingulate increased, suggesting that the default model network was changed in MCI patients. The increase in inferior temporal gyrus is consistent with previous studies which showed increased temporal activation in MCI and at-risk subjects relative to healthy controls [29, 43–46], but in our study the fact that the increase in the posterior cingulate is different from the previous report [15] maybe contributed to the different diagnosis criteria. The decreased ALFF in the medial frontal gyrus implies that the activities of neurons in this region were reduced in the early stage of MCI, and the excitability of its project fibers to the posterior regions was downregulated; the activities of the inferior temporal gyrus and posterior cingulate are compensatorily increased to keep the excitability of network and reinforce the relationship between the front gyrus and medium/posterior brain regions. However, in patients with AD, the neuronal activities are reduced due to the disruption of the compensatory increase in the posterior cingulate in resting state and thereby the brain regions associated with cognitive function like parahippocampus are affected [47].

It is generally agreed that acupuncture plays a homeostatic role and may have a greater effect on patients with a pathological imbalance compared to the healthy controls [48, 49]. Hence, imaging its effect on the brain networks in patients may further help to elucidate the mechanisms by which acupuncture achieves its therapeutic effects. KI3 is one of the most important acupoints for dementia. Previous studies demonstrated that acupuncture at KI3 with a depth of 1-2 cm enhanced the correlations related to the temporal regions in the poststimulus resting brain in MCI patients [31]. In the present study, we also found that acupuncture at KI3 could change more ALFF in MCI patients than that in healthy subjects. In MCI patients, acupuncture at KI3 increased ALFF in left parahippocampus, cingulate, and middle temporal gyrus of both sides and decreased it in precuneus. Those brain regions are components of the default model network. In MCI patients, acupuncture enhances the neuronal activity in the brain regions associated with cognitive function like parahippocampal gyrus and inhibits compensatory increase of activity in temporal regions like precuneus. The enhancement or inhibition of neuronal activities in above brain regions maintains the physiological homeostasis of default model network [7]. Interestingly, in healthy subjects, acupuncture did not significantly change neuronal activities in resting state. We speculated that in the healthy subjects the homeostasis of the neuronal activities in different brain regions is maintained well. In another study regarding the effect of acupuncture on ALFF in AD patients, we also found that acupuncture did not significantly affect the neuronal activities of the default network in rest state (data not shown). The efficiency of acupuncture on the default network may be reduced by the loss of neurons of brain regions associated with cognitive function. These data also give a hint that the early stage may be a “window of opportunity” to prevent dementia aggravation by acupuncture.

In the current study, we did not find the specific regularity from the difference between resting states with and without acupuncture at real or sham acupoint. Acupuncture, as a mechanic stimulation, may activate or inhibit one or one more brain region directly or may evoke or inhibit one brain region which affects other regions. This may be why we can see that the increase or decrease of neuronal activities induced by acupuncture at KI3 in some regions is similar to those induced by acupuncture at sham acupoint.

Specific effect of acupoints is an interesting issue which is helpful for the elucidation of the mechanism response for acupuncture. To verify the hypothesis that a specific region can be activated by acupuncture at an acupoint, we compared the activated brain regions by acupuncture at KI3 or sham acupoint and found that although acupuncture at either KI3 or sham acupoint can induce the increase or decrease of ALFF in the same brain region, but in some brain regions, a few of brain regions show different effects between acupuncture at KI3 and sham acupoint. Our results support the hypothesis that different acupoints can produce the different effects on some brain regions. Similar anatomical characteristics of KI3 and sham acupoint may be the reason that only few brain regions show different effects. To further elucidate the specific effect of acupoint, future studies should be directed to examine the effects of real and sham acupoints where anatomical characteristics are different significantly.

In general, we concluded that acupuncture at KI3 and sham acupoint improved the neuronal activities of the certain cognitive-related regions including medial frontal gyrus, inferior temporal gyrus, and posterior cingulate, which are components of default network. Acupuncture displays more significant effect on neuronal activities of the above brain regions in MCI patients than that in healthy controls. In MCI patients, acupuncture at KI3 presents different effects on the neuronal activities in middle frontal gyrus, superior temporal gyrus, and cingulate gyrus from those of acupuncture at sham acupoint and also exhibits different effects on a few of brain regions.

## Supplementary Material

In this supplementary material, three figures show statistical difference maps of ALFF in the resting state between acupuncture at KI3 and no acupuncture, or between with acupuncture at sham acupoint and no acupuncture or between acupuncture at KI3 and acupuncture at sham acupoint in normal control group. Three tables provide the changes in brain areas of ALFF signal in the rest state after acupuncture at KI3 or shame acupoint.

## Figures and Tables

**Table 1:** Characteristics of the MCI patients and normal controls.

Characteristics	MCI	Controls	*P* value
	(*n* = 7)	(*n* = 15)	
Age (years)	74.1 ± 7.8	70.2 ± 7.1	0.571
Education (years)	12.5 ± 3.1	11.4 ± 4.2	0.787
MMSE	27.0 ± 2.3	29.2 ± 1.3	*P* < 0.001
ADAS-cog	6.7 ± 2.9	2.5 ± 1.7	*P* < 0.001

MMSE: Mini-Mental State Examination (max = 30); ADAS: Alzheimer's Disease Assessment Scale.

**Table 2:** The brain areas that show significant difference in ALFF compared with the MCI patients with normal controls in the resting state.

Volume	Anatomical area	BA	H	*x*	*y*	*z*	Peak *t* value	Peak *P* value
3672	Medial frontal gyrus	BA6	L	4.5	22.5	68.5	−6.144	5.0 × 10^−4^
1539	Inferior temporal gyrus		R	−58.5	13.5	−21.5	5.1157	2.6 × 10^−4^
1242	Posterior cingulate		R	−22.5	58.5	20.5	5.0297	2.9 × 10^−4^

BA: Brodmann area; H: hemisphere; *x*: *x*-axis; *y*: *y*-axis; *z*: *z*-axis; L: left; and R: right.

**Table 3:** The brain areas that show significant difference in ALFF in MCI patients compared with normal controls in the resting state combined with acupuncture at KI3.

Volume	Anatomical area	BA	H	*x*	*y*	*z*	Peak *t* value	Peak *P* value
1107	Culmen		R	10.5	−40.5	−12.5	4.3581	9.3 × 10^−4^
972	Medial frontal gyrus anterior cingulate	10	R	10.5	37.5	−12.5	4.0723	1.6 × 10^−3^
918	Paracentral lobule		L	−4.5	−22.5	77.5	−5.436	1.5 × 10^−4^
891	Cerebellar tonsil Culmen		R	19.5	−34.5	−27.5	6.7392	2.1 × 10^−5^
675	Cuneus	23 18	L	−13.5	−73.5	8.5	4.7565	4.7 × 10^−4^

BA: Brodmann area; H: hemisphere; *x*: *x*-axis; *y*: *y*-axis; *z*: *z*-axis; L: left; and R: right.

**Table 4:** The brain areas that show significant difference in ALFF in MCI patients compared with normal controls in the resting state combined with acupuncture at sham acupoint.

Volume	Anatomical area	BA	H	*x*	*y*	*z*	Peak *t* value	Peak *P* value
1053	Medial frontal gyrus	6	L	−4.5	−25.5	74.5	−4.1004	1.5 × 10^−3^
972	Superior frontal gyrus	6	R	1.5	−4.5	68.5	−4.1896	1.3 × 10^−3^
	Medial frontal gyrus							

BA: Brodmann area; H: hemisphere; *x*: *x*-axis; *y*: *y*-axis; *z*: *z*-axis; L: left; and R: right.

**Table 5:** The brain areas of ALFF signal change significantly in MCI patients with acupuncture at KI3 compared with no acupuncture in the resting state.

Volume	Anatomical area	BA	H	*x*	*y*	*z*	Peak *t* value	Peak *P* value
1134	Parahippocampal gyrus	28	L	−13.5	−13.5	−9.5	5.644	1.3 × 10^−3^
972	Middle frontal gyrus		R	28.5	7.5	32.5	6.9909	4.2 × 10^−4^
891	Cingulate gyrus		L	−25.5	1.5	29.5	7.117	3.9 × 10^−4^
702	Cingulate gyrus	6	L	−25.5	−10.5	38.5	7.0511	4.1 × 10^−4^
	Middle frontal gyrus							
621	Subthalamic nucleus (thalamus)		R	10.5	−16.5	−3.5	9.6493	7.1 × 10^−5^

BA: Brodmann area; H: hemisphere; *x*: *x*-axis; *y*: *y*-axis; *z*: *z*-axis; L: left; and R: right.

**Table 6:** The brain areas of ALFF signal change significantly in MCI patients with acupuncture at sham acupoint compared with no acupuncture in the resting state.

Volume	Anatomical area	BA	H	*x*	*y*	*z*	Peak *t* value	Peak *P* value
864	Superior temporal gyrus	41	R	49.5	−31.5	11.5	−6.4773	6.5 × 10^−4^
864	Precentral gyrus	6	L	−46.5	−7.5	32.5	10.425	4.4 × 10^−5^
648	Medial frontal gyrus		R	13.5	34.5	35.5	12.005	2.0 × 10^−5^
594	Medial frontal gyrus	6	L	−10.5	−19.5	53.5	6.0288	9.5 × 10^−4^

BA: Brodmann area; H: hemisphere; *x*: *x*-axis; *y*: *y*-axis; *z*: *z*-axis; L: left; and R: right.

**Table 7:** The brain areas of ALFF signal change significantly in MCI patients with acupuncture at KI3 compared with acupuncture at sham acupoint in the resting state.

Volume	Anatomical area	BA	H	*x*	*y*	*z*	Peak *t* value	Peak *P* value
1593	Middle frontal gyrus	46	L	−43.5	19.5	20.5	−6.3792	7.0 × 10^−4^
864	Superior temporal gyrus	22	R	55.5	−28.5	5.5	15.064	5.4 × 10^−6^
594	Cingulate gyrus	31	R	7.5	−43.5	38.5	8.1928	1.8 × 10^−4^

BA: Brodmann area; H: hemisphere; *x*: *x*-axis; *y*: *y*-axis; *z*: *z*-axis; L: left; and R: right.
