# Supplementary material for: The Effects of Acupuncture at Real or Sham Acupoints on the Intrinsic Brain Activity in Mild Cognitive Impairment Patients
Source: Evid Based Complement Alternat Med. 2015 May 3;2015:529675. doi: 10.1155/2015/529675 (PMC4433670; doi:10.1155/2015/529675)

Supplementary materials

Figure S1 Two sample paired t-test shows statistical difference map of ALFF in the resting state between acupuncture at KI3 and no acupuncture in normal control group

Figure S2 Two sample paired t-test shows a statistical difference map of ALFF in the resting state between with acupuncture at sham acupoint and no acupuncture in normal control group

Figure S3 Two sample paired t-test shows a statistical difference map of ALFF in the resting state between acupuncture at KI3 and acupuncture at sham acupoint in normal control group

Table S1 The brain areas of ALFF signal change significantly in normal controls with acupuncture at KI3 compared with no acupuncture in the resting state

Volume	Anatomical area	BA	H	X	Y	Z	Peak *t* value	Peak *P* value
4239	paracentral lobule	5	R	1.5	-34.5	56.5	-6.3376	2.6×10^{-5}
756	cuneus	19	R	19.5	-88.5	26.5	-4.2657	9.1×10^{-4}
729	parahippocampal gyrus(lentiform nucleus)		R	16.5	-1.5	-6.5	4.6419	4.6×10^{-4}

BA: Brodman area; H: hemisphere; X: X axis; Y: Y axis; Z: Z axis; L: left; R: right.

Table S2 **The brain areas of ALFF signal change significantly in normal controls with acupuncture at sham acupoint compared with no acupuncture in the resting state**

Volume	Anatomical area	BA	H	X	Y	Z	Peak *t* value	Peak *P* value
4914	cuneus	19	R	16.5	-88.5	23.5	-6.499	3.0×10^{-5}
1674	paracentral lobule	4	R	4.5	-37.5	62.5	-4.9545	3.3×10^{-4}
864	anterior cingulate	24	L	-7.5	31.5	14.5	8.0612	3.5×10^{-6}
810	parahippocampal gyrus		L	-37.5	-25.5	-9.5	4.1217	1.4×10^{-3}
648	inferior frontal gyrus	47	R	16.5	7.5	-12.5	4.0115	1.7×10^{-3}
567	anterior cingulate	32	L	-4.5	19.5	-6.5	4.0575	1.6×10^{-3}

BA: Brodman area; H: hemisphere; X: X axis; Y: Y axis; Z: Z axis; L: left; R: right.

Table S3 **The brain areas of ALFF signal change significantly in normal controls with acupuncture at KI3 compared with acupuncture at sham acupoint in the resting state**

Volume	Anatomical area	BA	H	X	Y	Z	Peak *t* value	Peak *P* value
540	anterior cingulate	24<br>32	L	-1.5	28.5	-6.5	-3.7218	2.9×10^{-3}

BA: Brodman area; H: hemisphere; X: X axis; Y: Y axis; Z: Z axis; L: left; R: right.

Figure S1

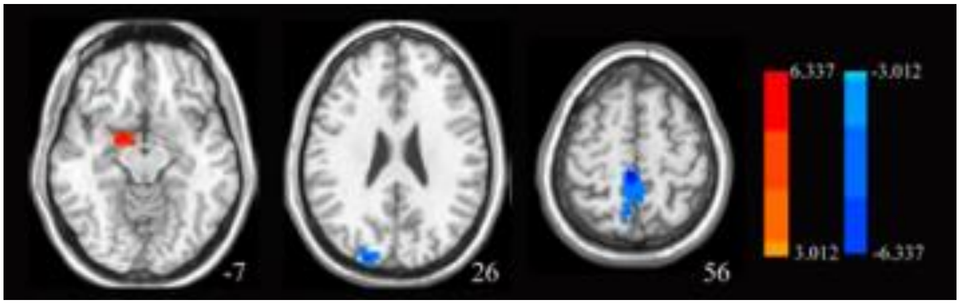

Figure S2

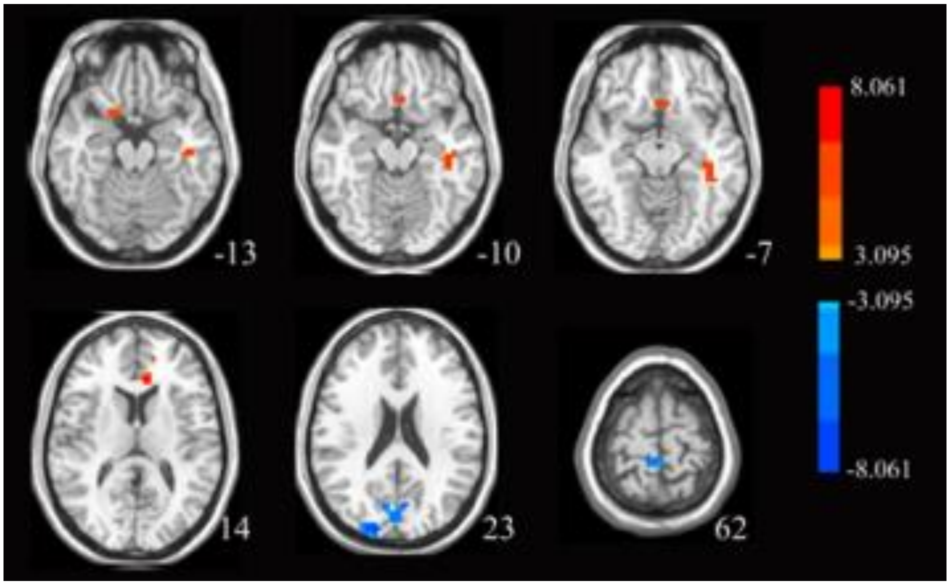

Figure S3

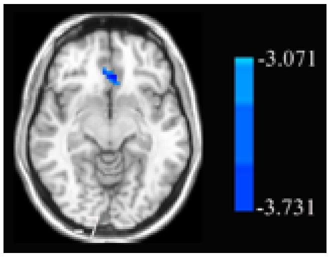

Supplement: Supplementary file 1 — In this supplementary material, three figures show statistical difference maps of ALFF in the resting state between acupuncture at KI3 and no acupuncture, or between with acupuncture at sham acupoint and no acupuncture or between acupuncture at KI3 and acupuncture at sham acupoint in normal control group. Three tables provide the changes in brain areas of ALFF signal in the rest state after acupuncture at KI3 or shame acupoint. [file 529675.f1.pdf]
